# Association between resident physicians from foreign medical schools and general medicine in-training examination scores: a nationwide cross-sectional study in Japan

**DOI:** 10.1186/s12909-026-08941-1

**Published:** 2026-03-03

**Authors:** Kosuke Ishizuka, Yuji Nishizaki, Miwa Sekine, Taro Shimizu, Kiyoshi Shikino, Yu Yamamoto, Sho Fukui, Masanori Nojima, Mitsuyasu Ohta, Yasuharu Tokuda

**Affiliations:** 1 Department of General Medicine, Yokohama City University School of Medicine, 3-9 Fukuura, Kanazawa-ku, Yokohama, Kanagawa pref. Japan; 2 Department of General Medicine, Yokohama City University Medical Center, Yokohama, Kanagawa Japan; 3 Division of Medical Education, Juntendo University School of Medicine, 2-1-1 Hongo Bunkyo-ku, Tokyo, Japan; 4 Department of Diagnostic and Generalist Medicine, Dokkyo Medical University Hospital, Tochigi, Japan; 5 Department of General Medicine, Chiba University Hospital, Chiba, Japan; 6 Department of Community-Oriented Medical Education, Graduate School of Medicine, Chiba University, Chiba, Japan; 7 Health Professional Development Center, Chiba University Hospital, Chiba, Japan; 8 Division of General Medicine, Center for Community Medicine, Jichi Medical University, Tochigi, Japan; 9 Department of Emergency and General Medicine, Kyorin University Hospital, Tokyo, Japan; 10 Center for Translational Research, The Institute of Medical Science Hospital, The University of Tokyo, Tokyo, Japan; 11 Muribushi Okinawa Center for Teaching Hospitals, Okinawa, Japan; 12 Tokyo Foundation for Policy Research, Tokyo, Japan

**Keywords:** Clinical training, GM-ITE, International medical graduates

## Abstract

**Background:**

This study aimed to evaluate the differences in test-based clinical knowledge between resident physicians who had received their undergraduate medical education in Japanese and foreign medical schools using the nationwide General Medicine In-Training Examination (GM-ITE^®^) scores and questionnaires.

**Method:**

We conducted a nationwide cross-sectional study of 9,106 resident physicians from 669 medical institutions in Japan who participated in the GM-ITE^®^ from January 17 to 30, 2024. The GM-ITE^®^ provides a highly reliable evaluation of resident physicians’ test-based clinical knowledge. The 2023 GM-ITE^®^ included 80 multiple-choice questions in four categories (Medical Interview and Professionalism, Symptomatology and Clinical Reasoning, Physical Examination and Clinical Skills, and Disease-Specific Topics), and six fields (Internal Medicine, Surgery, Pediatrics, Obstetrics and Gynecology, Psychiatry, and Emergency Medicine), with a maximum score of 80. Ten of the 80 questions were in English. We conducted a regional analysis (Japan, Non-Japan Asia, and Europe and Other) according to the local of the medical school where resident physicians received their undergraduate medical education.

**Result:**

The mean (standard deviation) GM-ITE^®^ scores were 43.2 (6.9) in the Japan group, 40.3 (4.9) in the Non-Japan Asia group, and 43.5 (6.4) in the Europe and Other group, and no statistically significant differences were observed by region (*p* = 0.153). The scores of resident physicians in the three regional groups did not differ significantly in the Medical Interview and Professionalism, Symptomatology and Clinical Reasoning, and Physical Examination and Clinical Skills categories, but those in the Non-Japan Asia group scored lower in the Disease-Specific Topics category (*p* = 0.003). The scores of the three regional groups did not differ significantly in the Internal Medicine (*p* = 0.637), Pediatrics (*p* = 0.296), Psychiatry (*p* = 0.112), and Emergency Medicine (*p* = 0.115) fields, but the Japan group scored higher in the Surgery (*p* = 0.007) and Obstetrics and Gynecology (*p* = 0.002) fields. The Europe and Other group scored significantly higher in the questions asked in English (*p* = 0.010).

**Conclusion:**

Overall GM-ITE^®^ scores showed no clear differences between groups; however, interpretation is limited by the small IMG sample, and category- or field-specific findings are exploratory. Observed differences warrant further study of educational and training background.

**Supplementary Information:**

The online version contains supplementary material available at 10.1186/s12909-026-08941-1.

## Introduction

As Japan becomes increasingly open to foreign cultures, the number of foreign immigrants continues to increase. Consequently, medical care in Japan is becoming more globalized, with patients representing a wider range of cultural and linguistic backgrounds [1–3]. In response to this shift, the demand for health professions who can navigate cultural and linguistic diversity in clinical settings has increased [1, 2]. Physicians who have studied medicine abroad bring valuable insights from international healthcare systems, introducing new perspectives and approaches that were previously uncommon in Japan [2, 4]. This diversity enhances patient-centered care, facilitates cross-cultural communication, and contributes to improving the overall quality of medical services [5, 6].

The Educational Commission for Foreign Medical Graduates (ECFMG) certificate is required for international medical graduates (IMG) to practice medicine in the United States [7]. In September 2010, the ECFMG announced that, from 2023 onwards, only graduates of medical schools accredited to international standards would be eligible to apply to the ECFMG [7–9]. In response to this announcement, medical education in Japan has rapidly developed to meet international standards [2, 7–10]. However, because medical education in Japan is mainly conducted in Japanese, opportunities to learn in multiple languages are limited [11]. Additionally, as the transition to competency-based medical education progresses, issues related to language ability and cross-cultural adaptation have arisen between those who have graduated from medical schools in Japan and those who have graduated from foreign medical schools. [12]. Therefore, the need for health professions with intercultural communication skills in Japan has increased. [12]. In addition, the number of foreign students studying at medical schools in Japan is very small [2, 8, 10].

Against this background, Japanese medical institutions are becoming more open to accepting doctors who have received their undergraduate medical education outside Japan [1, 2, 8, 10]. In Japan, these resident physicians may help address the needs of culturally and linguistically diverse patients and bring perspectives from different health systems, thereby enriching clinical practice and learning environments [1, 2, 5, 8, 10]. However, because residency training and patient care are conducted primarily in Japanese within a distinct clinical culture, it is important to compare their test-based performance with that of graduates of Japanese medical schools. Such comparisons can provide targeted educational support, fair assessment practices, and workforce planning as the number of these resident physicians increases.

Although language barriers, cultural adaptation, and educational differences among resident physicians educated outside Japan have been discussed, national-level evidence describing patterns of their test-based clinical knowledge with that of Japanese medical school graduates, particularly across specific clinical domains using a standardized in-training examination, remains limited [1, 9].

Resident physicians from foreign medical schools are likely to possess a globalized outlook and contribute to improving the quality of medical care through their interactions with patients from diverse nationalities and cultural backgrounds [6, 8, 9]. For this reason, the recruitment of such resident physicians is likely to increase in Japan in the future. However, when undergoing training in Japan’s unique culture and medical system, resident physicians who are IMG may encounter challenges in acquiring certain aspects of test-based clinical knowledge [2, 6, 13]. In order to train such resident physicians, the education system needs to not only deepen their understanding of the Japanese medical system, but also support their cross-cultural adaptation and language skills [14]. Therefore, clinical training supervisors need to understand the characteristics of resident physicians who are IMG and provide appropriate guidance [2, 6, 13].

The General Medicine In-Training Examination (GM-ITE^®^) is a nationwide standardized assessment aligned with Japan’s clinical training guidelines and is widely used to evaluate resident physicians’ test-based clinical knowledge across diverse training environments, making it an appropriate framework for comparing educational backgrounds within a uniform postgraduate training system. [15–18] Importantly, the GM-ITE^®^ does not aim to establish equivalence or superiority across groups, but rather provides a common assessment framework within which exploratory comparisons can be descriptively examined.

To examine whether educational and linguistic contexts are associated with performance patterns, undergraduate medical education was pragmatically grouped into Japan, Non-Japan Asia, and Europe and Other, based on similarities in educational systems and primary language of instruction [8, 19]. These regional categories were not intended to represent coherent or homogeneous educational systems, but were used as pragmatic analytic groupings to facilitate exploratory description of score distributions under a shared postgraduate training system.

This study aimed to explore whether resident physicians who completed undergraduate medical education outside Japan exhibit different patterns of performance on a nationwide in-training examination, within Japan’s standardized postgraduate training system.

## Methods

### Study design

We conducted a nationwide cross-sectional study to investigate how the test-based clinical knowledge of resident physicians who were IMG differed from those of graduates from Japanese medical schools. We examined the data of resident physicians who participated in the GM-ITE^®^ at the end of 2023 and explored the association between the GM-ITE^®^ score and the region in which resident physicians had received their undergraduate medical education. The reporting of the results adhered to the Strengthening the Reporting of Observational Studies in Epidemiology (STROBE) guidelines for cross-sectional studies [20].

### Context and participants

This study surveyed 9,106 resident physicians who worked at 669 medical institutions in Japan and participated in the GM-ITE^®^ program from January 17 to 30, 2024. Resident physicians who did not consent to participate in this study or for whom complete data were not available were excluded. Resident physicians who are IMG must pass the Japanese National Medical Practitioners Qualifying Examination and obtain a Japanese medical license before undergoing clinical training in Japan. This ensures that they are able to provide medical care communicating in Japanese and function in the Japanese medical environment [21]. In order to analyze the differences in test-based clinical knowledge between resident physicians who were IMG and those from Japanese medical schools, resident physicians from Japanese medical schools were only included if they were working at medical institutions with resident physicians who were IMG. This restriction was applied to improve comparability of the clinical training context and to reduce hospital-level confounding across institutions, including differences in educational resources and clinical exposure. However, this sampling strategy limits the representativeness of the Japan-trained cohort and may introduce selection bias.

### GM-ITE^®^

In the United States, the Residency Internal Medicine In-Training Examination (IM-ITE), is conducted to assess clinical knowledge during training. [22–24]. In 2011, the GM-ITE^®^, modeled on the IM-ITE, was developed and introduced in Japan. It has subsequently become a nationwide examination in Japan, with over half of all resident physicians participating. The GM-ITE^®^ is administered at the end of Postgraduate Year 1 (PGY-1) and Postgraduate Year 2 (PGY-2) and consists of questions aligned with the Ministry of Health, Labor and Welfare’s Clinical Training Guidelines for Physicians [15, 16]. It provides an objective, standardized assessment of resident physicians’ test-based performance aligned with Japan’s clinical training guidelines [15, 16]. It is organized into four categories: Medical Interview and Professionalism, Symptomatology and Clinical Reasoning, Physical Examination and Clinical Skills, and Disease-Specific Topics, and also includes fields related to the clinical rotation, including Internal Medicine, Surgery, Pediatrics, Obstetrics and Gynecology, Psychiatry, and Emergency Medicine. The GM-ITE^®^ is primarily a knowledge- and case-based examination; while it includes computer-based formats, such as video-based questions, that may reflect aspects of clinical practice, it does not directly assess workplace-based competencies such as bedside performance, communication, teamwork, or procedural skills. It aims to facilitate improvements in clinical training programs by providing both resident physicians and training program managers with an objective and reliable evaluation of resident physicians’ test-based clinical knowledge [17]. For resident physicians, the GM-ITE^®^ serves not as a pass-fail examination but rather as a means to identify proficiency in test-based clinical knowledge and weaknesses in specific clinical fields [18]. The 2023 GM-ITE^®^ comprised 80 multiple-choice questions, categorized as follows: Medical Interview and Professionalism (8 questions), Symptomatology and Clinical Reasoning (18 questions), Physical Examination and Clinical Skills (18 questions), and Disease-Specific Topics (36 questions). Each question was worth one point, resulting in a maximum score of 80. Although the primary language of the GM-ITE^®^ was Japanese, 10 out of the 80 questions were presented in English, to stress the importance of acquiring basic English proficiency in clinical practice.

### Data collection

Participants provided information about their resident level, region of undergraduate medical graduation, hospital location, type of hospital (community or university hospital), postgraduate year (PGY-1 or PGY-2), gender, average number of night-shifts per month, average number of inpatients, amount of self-study time per day, and number of duty-hours per week. After completing the GM-ITE^®^, participants were invited to complete a voluntary questionnaire, designed by the Japan Association for Medical Education Program (JAMEP). This questionnaire included items related to the residents’ training environment, including average number of night shifts per month, average number of inpatients, amount of self-study time per day, and number of duty-hours per week (Supplemental 1). The questionnaire items were based on factors previously identified as associated with GM-ITE^®^ scores [25, 26]. In addition to the overall score, the GM-ITE^®^ score was analyzed according to four categories (Medical Interview and Professionalism, Symptomatology and Clinical Reasoning, Physical Examination and Clinical Skills, and Disease-Specific Topics), and six fields (Internal Medicine, Surgery, Pediatrics, Obstetrics and Gynecology, Psychiatry, and Emergency Medicine). In addition, we conducted an analysis of the GM-ITE^®^ scores according to three regions (Japan, Non-Japan Asia, and Europe and Other) in which the resident physicians had received their undergraduate medical education. The Non-Japan Asia group was from the same Asian region as Japan, and most of the Europe and Other group had received their undergraduate medical education in English; therefore, we used these three broad regional groups to analyze how regional differences affected the GM-ITE^®^ score.

### Statistical analysis

Given the small number of resident physicians educated outside Japan, all analyses were exploratory and hypothesis-generating. First, we compared participants’ demographic and work-related characteristics according to the three regions in which the resident physicians had received their undergraduate medical education (Japan, Non-Japan Asia, and Europe and Other). The regional categories were defined pragmatically to reflect broad educational and linguistic contexts, and regional comparisons should be interpreted descriptively. Categorical variables were presented as frequencies and proportions and compared using chi-square tests. For contingency tables in which more than 20% of cells had expected counts below 5, we applied the Monte Carlo simulation method with 10,000 iterations to approximate p-values for the chi-square tests. Continuous variables were expressed as means and standard deviations (SDs) and one-way analysis of variance (ANOVA) was used to compare the scores between groups. Second, we assessed differences in test-based clinical knowledge, measured by the GM-ITE^®^ total score and its subdomains (by category and by field), across the three regions. One-way ANOVA was used to compare scores among regions, and post-hoc tests were conducted when appropriate. Finally, to investigate the associations between GM-ITE^®^ total scores and potential explanatory variables, including region of medical school graduation, we performed generalized linear models. The dependent variable was the GM-ITE^®^ total score. Fixed effects included region of graduation (reference: Japan), gender (reference: women), postgraduate year (PGY-1 or PGY-2), age group, number of night shifts per month, average number of assigned inpatients, amount of self-study time, and number of duty-hours per week. Language proficiency and cultural adaptation were not directly measured and were not included as covariates. Age was categorized into seven groups (24, 25, 26, 27, 28, 29, and ≥ 30 years) and was included to adjust for differences in age distribution across regions. A Gaussian distribution was assumed for the outcome. Results were reported as coefficients, 95% confidence intervals (CIs), and p-values for each variable. Because Japanese participants were sampled exclusively from hospitals where international medical graduates were also present, the primary analyses were conducted using generalized linear models without random effects (Table 3). To assess the robustness of the findings to potential within-hospital clustering, sensitivity analyses using mixed-effects models with a random intercept for hospital were additionally performed, and the results are presented in Supplemental 2. In addition, to further examine the robustness of the associations, we conducted additional analyses restricted to Japanese participants only, the results of which are presented alongside the overall analysis in Table 3.

### Ethics statement

This study was conducted in accordance with the principles of the Declaration of Helsinki, and the Ethical Guidelines for Medical and Health Research Involving Human Subjects. The research protocol was approved by the JAMEP Ethics Review Committee on December 21, 2023 (approval number: 23 − 16). All participants provided voluntary informed consent and were also given the option of withdrawing from the study.

## Results

### Participant characteristics

**Fig. 1:**
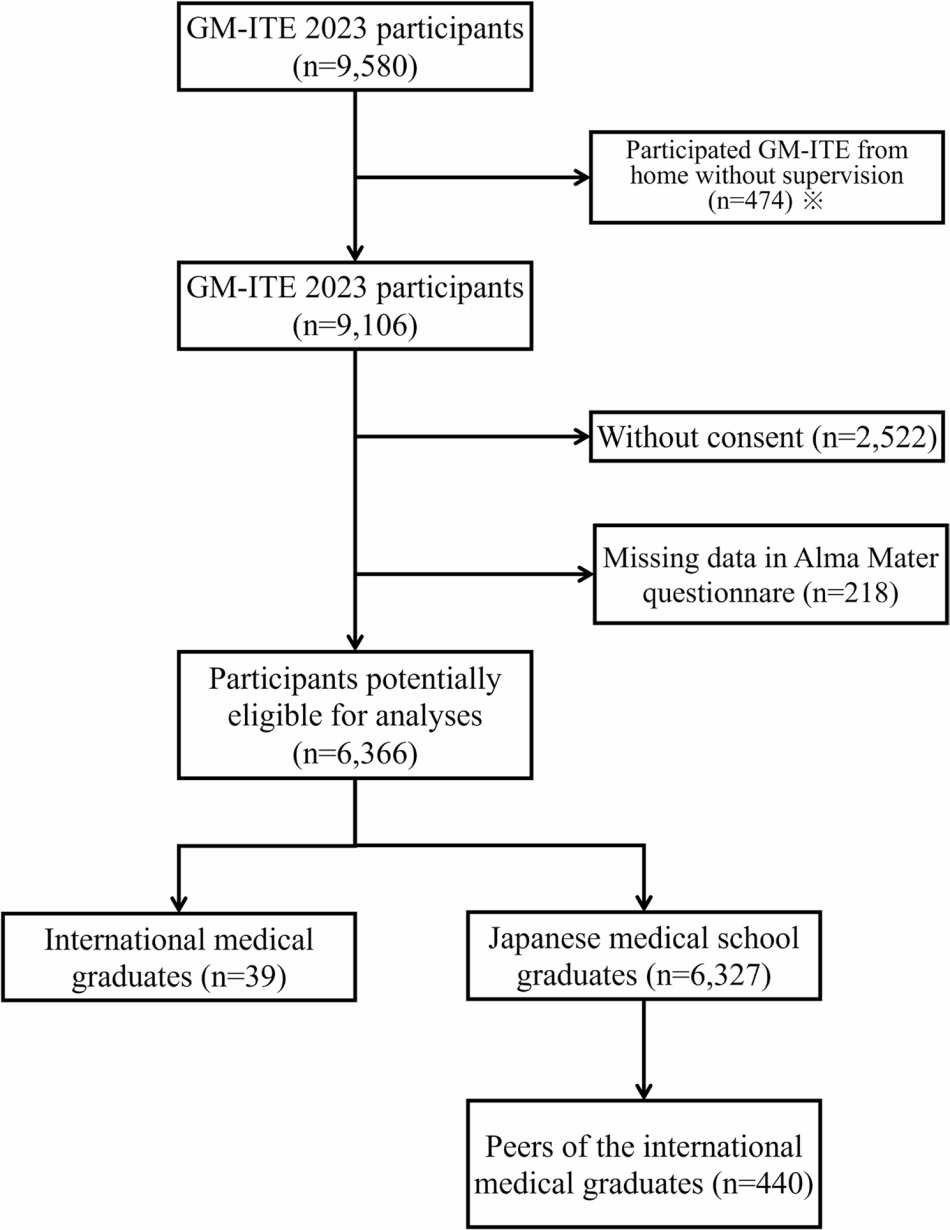
Flow diagram of the design. Japanese medical school graduates were included only from hospitals where at least one resident physician who completed undergraduate medical education outside Japan was present

Overall, 9,106 resident physicians from 669 institutions participated in the GM-ITE^®^, of which 6,366 resident physicians from 655 institutions gave consent (response rate: 69.9%). Of these, 22 and 17 resident physicians were in the Non-Japan Asia, and Europe and Other groups, respectively. For the Japan group, the survey target was resident physicians who were enrolled in the same medical institution as resident physicians who were IMG; therefore, consistent with this sampling strategy, 440 resident physicians were included in the final analysis (Fig. 1). Table 1 shows the baseline characteristics of the resident physicians in this study. Among the participants in this study, PGY-1 was 234 (53.2%) in the Japan group, 13 (59.1%) in the Non-Japan Asia group, and 12 (70.6%) in the Europe and Other group. The proportion of female participants was significantly higher in the Non-Japan Asia group (50.0%) and Europe and Other group (70.6%) than in the Japan group (34.3%) (*p* = 0.004). In the Japan group, the largest proportion of respondents was aged 26 years (29.8%), but in the Non-Japan Asia group (72.7%) and Europe and Other group (41.2%), the largest proportion was aged 30 years and over (*p* < 0.001). The hospital location was rural the Japan group (44.1%), Non-Japan Asia group (45.5%), and Europe and Others group (52.9%). The proportion of participants affiliated with a community hospital was 40.9% in the Japan group, 50.0% in the Non-Japan Asia group, and 64.7% in the Europe and Other group. Many participants had worked 3 to 5 night shifts per month. Among the participants, the modal number of assigned inpatients in the Japan group was 5 to 9, whereas the modal number of assigned inpatients in the Non-Japan Asia and Europe and Other groups was 0 to 4. In all three groups, the modal amount of self-study times was 1 to 30 min per day, and more than half of the participants worked less than 60 duty-hours per week. The mean GM-ITE^®^ scores of the participants were 43.2 (SD: 6.9) in the Japan group, 40.3 (SD: 4.9) in the Non-Japan Asia group, and 43.5 (SD: 6.4) in the Europe and Other group, with no statistically significant difference among groups (*p* = 0.153).

**Table 1:** Baseline characteristics of the resident physicians

Resident-level information	Japan (*N* = 440)	Non-Japan Asia (*N* = 22)	Europe and Others (*N* = 17)	*p*-value
Gender				*p* = 0.004
Men, n (%)	289 (65.7)	11 (50.0)	5 (29.4)	
Women, n (%)	151 (34.3)	11 (50.0)	12 (70.6)	
Missing, n (%)	0 (0.0)	0 (0.0)	0 (0.0)	
Grade				*p* = 0.328
PGY-1, n (%)	234 (53.2)	13 (59.1)	12 (70.6)	
PGY-2, n (%)	206 (46.8)	9 (40.9)	5 (29.4)	
Missing, n (%)	0 (0.0)	0 (0.0)	0 (0.0)	
Age				*p* < 0.001
24 years old, n (%)	17 (3.9)	0 (0.0)	0 (0.0)	
25 years old, n (%)	82 (18.6)	0 (0.0)	0 (0.0)	
26 years old, n (%)	131 (29.8)	0 (0.0)	0 (0.0)	
27 years old, n (%)	77 (17.5)	0 (0.0)	3 (17.6)	
28 years old, n (%)	54 (12.3)	3 (13.6)	5 (29.4)	
29 years old, n (%)	17 (3.9)	3 (13.6)	2 (11.8)	
Older than 30 years old, n (%)	61 (13.9)	16 (72.7)	7 (41.2)	
Missing, n (%)	1 (0.2)	0 (0.0)	0 (0.0)	
Location				*p* = 0.768
Rural, n (%)	194 (44.1)	10 (45.5)	9 (52.9)	
Urban, n (%)	246 (55.9)	12 (54.5)	8 (47.1)	
Missing, n (%)	0 (0.0)	0 (0.0)	0 (0.0)	
Type of hospital				*p* = 0.112
Community Hospital, n (%)	180 (40.9)	11 (50.0)	11 (64.7)	
University Hospital, n (%)	260 (59.1)	11 (50.0)	6 (35.3)	
Missing, n (%)	0 (0.0)	0 (0.0)	0 (0.0)	
Night shifts per month				*p* = 0.383
0, n (%)	38 (8.6)	3 (13.6)	2 (11.8)	
1–2, n (%)	103 (23.4)	7 (31.8)	1 (5.9)	
3–5, n (%)	278 (63.2)	10 (45.5)	13 (76.5)	
≥ 6, n (%)	17 (3.9)	2 (9.1)	1 (5.9)	
Unknown, n (%)	1 (0.2)	0 (0.0)	0 (0.0)	
Missing, n (%)	3 (0.7)	0 (0.0)	0 (0.0)	
Average number of assigned inpatients				*p* = 0.306
0–4, n (%)	129 (29.3)	11 (50.0)	8 (47.1)	
5–9, n (%)	180 (40.9)	6 (27.3)	8 (47.1)	
10–14, n (%)	90 (20.5)	4 (18.2)	1 (5.9)	
≥ 15, n (%)	26 (5.9)	1 (4.5)	0 (0.0)	
Unknown, n (%)	10 (2.3)	0 (0.0)	0 (0.0)	
Self-study time per day (minutes)				*p* = 0.737
0, n (%)	12 (2.7)	0 (0.0)	0 (0.0)	
1–30, n (%)	222 (50.5)	11 (50.0)	7 (41.2)	
31–60, n (%)	156 (35.5)	7 (31.8)	7 (41.2)	
61–90, n (%)	37 (8.4)	3 (13.6)	2 (11.8)	
≥ 91, n (%)	7 (1.6)	1 (4.5)	1 (5.9)	
Missing, n (%)	6 (1.4)	0 (0.0)	0 (0.0)	
Duty-hours per week (hours)				*p* = 0.617
< 45, n (%)	36 (8.2)	0 (0.0)	2 (11.8)	
≥ 45–<50, n (%)	92 (20.9)	6 (27.3)	4 (23.5)	
≥ 50–<55, n (%)	87 (19.8)	3 (13.6)	2 (11.8)	
≥ 55–<60, n (%)	57 (13.0)	3 (13.6)	3 (17.6)	
≥ 60–<65, n (%)	46 (10.5)	4 (18.2)	0 (0.0)	
≥ 65–<70, n (%)	31 (7.0)	2 (9.1)	1 (5.9)	
≥ 70–<80, n (%)	36 (8.2)	1 (4.5)	4 (23.5)	
≥ 80–<90, n (%)	26 (5.9)	3 (13.6)	1(5.9)	
≥ 90–<100, n (%)	11 (2.5)	0 (0.0)	0 (0.0)	
≥ 100, n (%)	9 (2.0)	0 (0.0)	0 (0.0)	
Missing, n (%)	9 (2.0)	0 (0.0)	0 (0.0)	

Percentages were calculated using the total sample size within each group (Japan group: *N* = 440; Non-Japan Asia group: *N* = 22; Europe and Others group: *N* = 17)

Table 2 shows the results for GM-ITE^®^ by category and field of practice. The results did not differ significantly between the three regional groups in the Medical Interview and Professionalism (*p* = 0.763), Symptomatology and Clinical Reasoning (*p* = 0.334), Physical Examination and Clinical Skills (*p* = 0.373) categories. However, a statistically significant difference among the three regional groups was observed in the Disease-Specific Topics category (*p* = 0.003), with higher mean scores in the Japan and Europe and Other groups than in the Non-Japan Asia group. The results did not differ significantly between the three regional groups in the fields of Internal Medicine (*p* = 0.637), Pediatrics (*p* = 0.296), Psychiatry (*p* = 0.112), and Emergency Medicine (*p* = 0.115), whereas, statistically significant differences among the three regional groups were observed in the Surgery (*p* = 0.007) and Obstetrics and Gynecology (*p* = 0.002) fields, with the Japan group having a higher on the GM-ITE^®^ score. The three groups also showed statistically significant differences in the scores for questions asked in English (*p* = 0.010).

**Table 2:** The results for GM-ITE^®^ by field and by area of practice

Resident-level information	Japan (*N* = 440)	Non-Japan Asia (*N* = 22)	Europe and Others (*N* = 17)	*p*-value
Total Score (Mean, SD)	43.2 ± 6.9	40.3 ± 4.9	43.5 ± 6.4	*p* = 0.153
By Category				
Medical Interview and Professionalism (Mean, SD)	5.6 ± 1.2	5.6 ± 1.3	5.4 ± 1.3	*p* = 0.763
Symptomatology & Clinical Reasoning (Mean, SD)	9.6 ± 2.3	9.1 ± 2.1	10.1 ± 2.0	*p* = 0.334
Physical Examination & Clinical Skills (Mean, SD)	8.1 ± 2.3	8.4 ± 2.3	8.8 ± 1.9	*p* = 0.373
Disease Specifics (Mean, SD)	19.9 ± 3.6	17.3 ± 2.7	19.1 ± 4.2	*p* = 0.003
By Field				
Internal Medicine (Mean, SD)	19.0 ± 4.1	18.3 ± 3.0	19.4 ± 4.0	*p* = 0.637
Surgery (Mean, SD)	4.9 ± 1.3	4.1 ± 1.6	4.2 ± 1.1	*p* = 0.007
Pediatrics (Mean, SD)	2.6 ± 1.0	2.7 ± 0.8	3.0 ± 1.0	*p* = 0.296
Obstetrics & Gynecology (Mean, SD)	2.8 ± 0.9	2.1 ± 0.8	2.5 ± 0.9	*p* = 0.002
Psychiatry (Mean, SD)	2.6 ± 0.9	2.2 ± 1.0	2.5 ± 0.9	*p* = 0.112
Emergency Medicine (Mean, SD)	5.6 ± 1.7	5.3 ± 1.3	6.4 ± 1.5	*p* = 0.115
Questions asked in English (Mean, SD)	3.7 ± 1.8	3.4 ± 1.5	4.9 ± 1.3	*p* = 0.010

Table 3 shows the relationship between the GM-ITE^®^ score and resident-level information using multivariable analysis. All estimates were adjusted for age, gender, postgraduate year, number of night shifts per month, average number of assigned inpatients, self-study time, and duty-hours per week, and statistically significant associations were observed for postgraduate year (*p* = 0.011), age (*p* = 0.048), night shifts per month (*p* = 0.047), and average number of assigned inpatients (*p* < 0.001 to *p* = 0.013).

**Table 3:** Multivariable analysis of factors associated with GM-ITE^®^ total scores in the overall sample and in Japanese participants only

Factors	Overall analysis (*n* = all)		Japan-only analysis (*n* = 440)	
	Adjusted estimated coefficient (95%CI)	*p*-value	Adjusted estimated coefficient (95%CI)	*p*-value
Region of Medical School Graduation				
Japan	Reference			
Non-Japan Asia	−1.27 (−4.28 to 1.74)	*p* = 0.408		
Europe and Others	1.25 (−2.04 to 4.55)	*p* = 0.455		
Gender				
Women	Reference		Reference	
Men	0.57 (−0.70 to 1.85)	*p* = 0.378	−0.49 (−1.84 to 0.85)	*p* = 0.471
Grade				
PGY-1	Reference		Reference	
PGY-2	1.73 (0.41 to 3.05)	*p* = 0.011	1.84 (0.45 to 3.24)	*p* = 0.010
Age				
24 years old	Reference		Reference	
25 years old	1.87 (−1.53 to 5.26)	*p* = 0.280	1.98 (−1.45 to 5.41)	*p* = 0.257
26 years old	0.18 (−3.17 to 3.53)	*p* = 0.917	0.16 (−3.23 to 3.55)	*p* = 0.927
27 years old	−2.51 (−6.02 to 0.99)	*p* = 0.159	−2.75 (−6.30 to 0.81)	*p* = 0.130
28 years old	−1.91 (−5.48 to 1.65)	*p* = 0.292	−1.96 (−5.61 to 1.69)	*p* = 0.291
29 years old	−1.81 (−6.08 to 2.46)	*p* = 0.405	−2.33 (−6.87 to 2.21)	*p* = 0.314
Older than 30 years old	−3.57 (−7.11 to −0.04)	*p* = 0.048	−3.20 (−6.83 to 0.42)	*p* = 0.083
Night shifts per month				
0	Reference		Reference	
1–2	1.66 (−0.70 to 4.01)	*p* = 0.168	1.95 (−0.55 to 4.45)	*p* = 0.126
3–5	1.13 (−1.07 to 3.34)	*p* = 0.312	1.57 (−0.79 to 3.93)	*p* = 0.191
≥ 6	3.64 (0.06 to 7.21)	*p* = 0.047	4.08 (0.19 to 7.97)	*p* = 0.040
Unknown	−2.47 (−15.30 to 10.36)	*p* = 0.705	−2.35 (−15.33 to 10.64)	*p* = 0.723
Average number of assigned inpatients				
5–9	Reference		Reference	
0–4	−0.45 (−1.88 to 0.98)	*p* = 0.535	−0.21 (−1.73 to 1.30)	*p* = 0.781
10–14	−3.32 (−4.92 to −1.72)	*p* < 0.001	−3.47 (−5.12 to −1.81)	*p* < 0.001
≥ 15	−3.41 (−6.09 to −0.72)	*p* = 0.013	−3.37 (−6.14 to −0.60)	*p* = 0.017
Unknown	−3.05 (−7.41 to 1.31)	*p* = 0.170	−3.18 (−7.60 to 1.24)	*p* = 0.158
Self-study time per day (minutes)				
0	Reference		Reference	
1–30	−0.59 (−4.38 to 3.20)	*p* = 0.760	−0.58 (−4.41 to 3.25)	*p* = 0.767
31–60	1.73 (−2.11 to 5.57)	*p* = 0.377	2.02 (−1.87 to 5.91)	*p* = 0.308
61–90	2.38 (−1.80 to 6.56)	*p* = 0.263	2.78 (−1.48 to 7.04)	*p* = 0.201
≥ 91	3.22 (−2.37 to 8.82)	*p* = 0.258	3.56 (−2.50 to 9.61)	*p* = 0.249
Duty-hours per week (hours)				
< 45, n (%)	Reference		Reference	
≥ 45–<50, n (%)	1.23 (−1.25 to 3.71)	*p* = 0.330	1.21 (−1.39 to 3.82)	*p* = 0.361
≥ 50–<55, n (%)	0.62 (−1.94 to 3.17)	*p* = 0.635	0.48 (−2.18 to 3.15)	*p* = 0.723
≥ 55–<60, n (%)	1.98 (−0.72 to 4.68)	*p* = 0.151	1.82 (−1.01 to 4.65)	*p* = 0.207
≥ 60–<65, n (%)	2.77 (−0.12 to 5.66)	*p* = 0.061	2.79 (−0.23 to 5.81)	*p* = 0.070
≥ 65–<70, n (%)	0.79 (−2.36 to 3.94)	*p* = 0.623	1.04 (−2.28 to 4.36)	*p* = 0.539
≥ 70–<80, n (%)	1.88 (−1.11 to 4.88)	*p* = 0.217	1.90 (−1.27 to 5.06)	*p* = 0.240
≥ 80–<90, n (%)	1.94 (−1.30 to 5.18)	*p* = 0.241	1.66 (−1.78 to 5.10)	*p* = 0.344
≥ 90–<100, n (%)	0.28 (−4.17 to 4.72)	*p* = 0.903	−0.12 (−4.65 to 4.42)	*p* = 0.960
≥ 100, n (%)	3.12 (−1.71 to 7.95)	*p* = 0.205	3.14 (−1.79 to 8.06)	*p* = 0.211

In addition, sensitivity analyses using mixed-effects models with a random intercept for hospital are presented in Supplemental 2. Although statistical significance differed for some variables, the estimated coefficients were similar in direction and magnitude to those observed in the primary analysis.

## Discussion

### Overall GM-ITE^®^ score among the three groups

In this study, the overall GM-ITE^®^ score did not differ significantly between the Japan, Non-Japan Asia, and Europe and Other regional groups. However, given the extremely small number of resident physicians educated outside Japan and limited statistical power, non-significant findings should be interpreted with caution and not overinterpreted as evidence of equivalence between groups. In addition, given that multiple subgroup comparisons were performed without multiplicity adjustment, statistically significant findings in these sub-analyses may warrant cautious interpretation and are better regarded as exploratory or hypothesis-generating. Clinical training hospitals in Japan are run to uniform standards nationwide, and clinical education curricula and clinical training guidance systems are standardized, so the education of resident physicians is relatively consistent [10]. However, the differences in medical education culture between the Non-Japan Asia and the Europe and Other groups could be one possible explanation for differences in how resident physicians adapt to working in a Japanese medical environment. Although medical education in Japan is highly standardized and maintains an educational style that emphasizes basic knowledge, medical education systems vary widely, and such differences cannot be directly assessed in the present study; therefore, references to educational style or cultural background are based on prior literature rather than empirical evidence from our data [8, 14, 27]. Because language proficiency, cross-cultural adaptation, and field-specific clinical exposure were not directly measured, the present study cannot determine whether or how these factors contributed to the absence of clear differences in overall GM-ITE^®^ scores. Accordingly, this finding should be interpreted descriptively, and future studies incorporating direct measures of these factors are warranted to explore potential mechanisms. In the Europe and Other group, experience of education centered on English may have made it easier to respond to the English-language questions [2]. In interpreting these findings, GM-ITE^®^ scores reflect resident physicians’ test-based clinical knowledge rather than comprehensive clinical competence, as key domains such as bedside decision-making, communication, professionalism, teamwork, procedural skills, and cultural competence are not directly assessed.

### Significant differences in the disease-specific topics category

In the Disease-Specific Topics category, the scores of the Japanese group of resident physicians were higher than those of the other groups. Given the small number of resident physicians educated outside Japan, this between-group difference should be interpreted cautiously and treated as exploratory rather than explanatory. In Japan, medical schools still provide systematic medical education from the basic to the clinical level, and this fosters in-depth knowledge of specific diseases [1, 28–30]. However, the present study does not permit definitive attribution of subgroup differences to specific educational structures, policies, or country-level curricular characteristics. Future studies using larger and more balanced samples are needed to directly examine these factors. Furthermore, differences in the number of patients seen by resident physicians may also be relevant; however, because field-specific clinical exposure was not directly measured in this study, this observation should be considered hypothesis-generating.

### High scores in the Japan group in the surgery and obstetrics and gynecology fields

In the Surgery and Obstetrics and Gynecology fields, the Japan group of resident physicians demonstrated higher GM-ITE^®^ scores than the other groups. Given the small number of resident physicians educated outside Japan, these field-specific differences should be interpreted cautiously and regarded as exploratory. Prior literature has described that undergraduate and postgraduate medical education in Japan places substantial emphasis on foundational knowledge and structured learning opportunities in surgical and obstetric fields, and that medical students are exposed to standardized curricula covering specific techniques and clinical processes from an early stage [1, 9, 10, 31, 32]. These descriptions provide contextual background on Japanese medical education; however, the present study was not designed to evaluate educational content, clinical exposure, or language-related factors, and therefore cannot determine whether or how such characteristics relate to the observed score differences.

### Comparison of scores in the symptomatology and clinical reasoning, and physical examination and clinical skills categories, and the emergency medicine field

One possible explanation for the absence of significant differences in these categories and the Emergency Medicine field is that such competencies are commonly acquired through hands-on clinical experience during residency training [29]. In the clinical training system in Japan, resident physicians are provided with opportunities to directly deal with a wide variety of cases, and they are encouraged to gain practical experience [10]. It is possible that shared exposure to practical clinical training during residency contributes to a relatively uniform distribution of scores across these domains; however, this interpretation remains speculative, as the extent and nature of clinical experience were not directly measured in this study [2, 5]. Resident physicians need to acquire hands-on experience, and previous studies have also shown a positive correlation between the average number of assigned inpatients and the resident physicians’ GM-ITE^®^ score [25]. The results of the multivariable analysis in this study also showed that resident physicians who had 10 or more average number of assigned inpatients had higher GM-ITE^®^ scores than those who had 0 to 9 average number of assigned inpatients. This result can be summarized in the astute words of Sir William Osler: “Studying disease without books is like sailing in uncharted waters, while studying books without patients is like not going to sea at all” [33, 34].

### High scores in the Europe and other group in the questions asked in english

The high scores in the Europe and Other group in the questions asked in English may be because many of them have experience of using English as their main language of education [8]. They are accustomed to accessing medical education and literature in English, so they may have felt less resistance to the questions asked in English, which may have led to their high scores.

### Influence of language barriers and cultural background

Language barriers and cultural background may be relevant, but they were not directly measured, and any discussion of their influence on GM-ITE^®^ performance is therefore contextual and hypothesis-generating rather than causal [5, 35]. Resident physicians from the Non-Japan Asia and Europe and Other groups sometimes find it challenging to communicate in Japanese and adapt to the medical culture [6, 8, 35]. However, if these trainees have the ability to adapt to a multicultural environment and multilingual skills, their adaptation to the Japanese medical environment may be facilitated, which may support learning and clinical performance [6, 8, 35]. Recent educational research also highlights the value of instructional strategies such as simulation-based and flipped classroom learning that promote active, reflective, and experiential engagement, which may help learners integrate knowledge and skills in complex clinical and cultural contexts [36]. In Japanese medical education, although a standardized curriculum has been developed, particularly for clinical training, it has been pointed out that the language barrier still affects the understanding and practice of the educational content. Urushibara-Miyachi et al. state that when adapting international educational concepts to the Japanese context, reconstructing terminology that is appropriate to the context of medical education, rather than simply translating it, is important [12]. This process should take the cultural and social context into account to deepen the learners’ understanding and promote the acquisition of the competencies that resident physicians need to acquire [12]. Furthermore, Nishigori [14]. emphasizes that the language barrier in Japanese medical education is a particularly significant issue, and that multicultural adaptability is essential. For example, whereas resident physicians from Europe and Other, whose primary language is English, have difficulty adapting to the medical environment in Japan, their access to English-language resources and international educational backgrounds are thought to complement their adaptability [14].

### Recommendations for improving the quality of clinical training based on this research

Given the exploratory nature of this study and the imprecision caused by sample imbalance, category- and field-specific differences should be interpreted with caution, and no definitive conclusions regarding underlying educational or cultural mechanisms can be drawn. Instead, these findings may inform future research directions and the design of pragmatic, non-assumptive educational supports. Furthermore, the quality of clinical training also needs to be improved by enhancing language support and educational programs for cross-cultural adaptation of resident physicians who are IMG [6, 10]. From an educational perspective, these findings suggest pragmatic supports for resident physicians who completed undergraduate medical education outside Japan, including structured onboarding to the Japanese medical system, tailored Japanese-language support, and longitudinal mentorship to facilitate cultural adaptation and help-seeking. Instructional approaches grounded in active, reflective, and experiential learning—such as simulation-based and flipped classroom learning—may also enhance learners’ integration of clinical knowledge and adaptability in multicultural training environments [36]. Such supports may be particularly relevant for domains in which score differences were observed, including Disease-Specific Topics, Surgery, and Obstetrics and Gynecology, where targeted learning resources and supervised clinical exposure could be prioritized. These approaches are consistent with prior IMG literature emphasizing orientation, mentorship, and supportive learning environments during transition to host-country practice [19]. In addition, future assessment strategies could enhance program evaluation by complementing the GM-ITE^®^ with competency- or performance-based measures, such as workplace-based assessments and multisource feedback, to capture domains not assessed by written examinations, including communication, professionalism, teamwork, and clinical performance in context [37, 38]. This complementary approach may help identify specific support needs for IMG resident physicians beyond test-based performance and monitor longitudinal improvement.

### Limitations

This study has several limitations. First, this study is limited by a substantial imbalance in sample size across regions, particularly the small number of resident physicians who graduated from non-Japanese medical schools. Although this reflects the real-world distribution of international medical graduates in Japan, it may limit generalizability, reduce statistical power to detect modest between-group differences, and result in imprecise effect estimates, with some differences potentially over- or underestimated. In addition, because resident physicians from Japanese medical schools were included only from hospitals that also employed resident physicians educated outside Japan, the Japanese resident physicians in this study may not represent the broader population of Japanese resident physicians, and this sampling strategy may limit generalizability despite improving comparability of training environments. In particular, this imbalance increases the likelihood of Type II error; therefore, non-significant findings should not be interpreted as evidence of equivalence. Future studies with larger, more balanced samples are needed to improve statistical power and enable more precise estimation of between-group differences. Second, the category-, field-, and English-item subgroup analyses involved multiple statistical comparisons, and no formal adjustment for multiplicity was applied. As a result, the possibility of Type I error cannot be excluded, and statistically significant findings from these sub-analyses should be interpreted cautiously and regarded as exploratory and hypothesis-generating rather than confirmatory. Third, the GM-ITE^®^ is a written in-training examination that primarily assesses cognitive knowledge and test-based performance and does not directly evaluate key workplace-based competencies such as real-time clinical decision-making, communication, teamwork, or procedural skills; therefore, nonsignificant differences in GM-ITE^®^ scores should not be interpreted as equivalence in overall clinical practice. Future studies should incorporate workplace-based assessments and multisource feedback to provide a more comprehensive evaluation of resident physicians’ clinical competence and educational needs [37, 38]. Fourth, the data used in this study are based primarily on information that was self-reported at the end of the GM-ITE^®^. Although self-reported data provide a comprehensive overview, they may contain bias or inaccuracies, and the reported experience and actual experience may differ. Fifth, this study is limited in its ability to apply the results to resident physicians in other countries because it was limited to resident physicians undergoing clinical training in Japan. In addition, as the sample was limited to a specific population, the generalizability of the results may be limited. Regarding the country in which IMG received their medical education, the analysis was conducted by broad Non-Japan Asia, and Europe and Other regional categories, and the distribution of the results by country was not considered. The quality of medical school education may differ not only by region but also by country, and the extent to which the differences from Japanese medical school education can be generalized may be limited. Therefore, these regional categories should be interpreted as pragmatic groupings rather than as homogeneous educational systems, and the comparisons across regions should be interpreted with caution. Sixth, because the study only included resident physicians who participated in the voluntary GM-ITE^®^ program, there is a possibility of selection bias. In particular, the resident physicians who participated in this study were approximately one-third of all resident physicians in Japan, and many of the hospitals surveyed were facilities that were enthusiastic about education. Therefore, the results might not be representative of all resident physicians. Seventh, improvements and changes in clinical training programs were not considered. The results of this study reflect the situation at a specific timepoint, and the educational outcomes may change due to future feedback and policy changes. Eighth, some potential confounding factors that may affect the resident physicians’ experience and GM-ITE^®^ scores were not examined. For example, the number of supervisors at the clinical training hospital, the number of years of clinical experience of the supervisors, and the content of the educational program may have affected the resident physicians’ experience and GM-ITE^®^ scores. Ninth, this study did not collect information on the nationality of the resident physicians or the country in which they grew up before entering medical school. This is important when considering the influence of cultural background and language and is an issue for future consideration. Finally, the response rate is also one of the limitations of this study. In this study, resident physicians from Japanese medical schools were included in the survey if they were working at institutions with resident physicians from foreign medical schools, and this may limit the generalizability of the results.

## Conclusion

In this study, no consistent differences were observed in the overall GM-ITE^®^ scores between resident physicians who are IMG and those from Japanese medical schools. While category- and field-specific differences were observed, these findings should be interpreted with caution given the exploratory nature of the study. Understanding the characteristics of resident physicians who are IMG remains an important area for future research, and larger studies with more balanced samples are needed to further examine potential sources of variation.

## Supplementary Information

Supplementary Material 1.

Supplementary Material 2.

## Data Availability

Resident physicians who joined in this study did not obtain consent for their data to be shared publicly, so supporting data is not available. The corresponding author will respond to inquiries on the data analyses in this study.
