# Supplementary material for: Association between resident physicians from foreign medical schools and general medicine in-training examination scores: a nationwide cross-sectional study in Japan
Source: BMC Med Educ. 2026 Mar 3;26:571. doi: 10.1186/s12909-026-08941-1 (PMC13064377; doi:10.1186/s12909-026-08941-1)
Supplement: Supplementary file 1 — Supplementary Material 1. [file 12909_2026_8941_MOESM1_ESM.docx]

**Supplemental 1:** Questionnaire about resident physicians' training environment.

**1. Average number of night shifts per month**

(1) 0

(2) 1-2

(3) 3-5

(4) ≥ 6

(5) Unknown

**2. Average number of assigned inpatients**

(1) 0-4

(2) 5-9

(3) 10-14

(4) ≥ 15

(5) Unknown

**3. Amount of self-study time per day (minutes)**

(1) 0

(2) 1-30

(3) 31-60

(4) 61-90

(5) ≥ 91

**4. Number of duty-hours per week (hours)**

(1) < 45

(2) ≥ 45-< 50

(3) ≥ 50-< 55

(4) ≥ 55-< 60

(5) ≥ 60-< 65

(6) ≥ 65-< 70

(7) ≥ 70-< 80

(8) ≥ 80-< 90

(9) ≥ 90-< 100

(10) ≥ 100
