# Supplementary material for: Association between resident physicians from foreign medical schools and general medicine in-training examination scores: a nationwide cross-sectional study in Japan
Source: BMC Med Educ. 2026 Mar 3;26:571. doi: 10.1186/s12909-026-08941-1 (PMC13064377; doi:10.1186/s12909-026-08941-1)
Supplement: Supplementary file 2 — Supplementary Material 2. [file 12909_2026_8941_MOESM2_ESM.docx]

**Supplemental 2.** Sensitivity analyses using mixed-effects models with a random intercept for hospital. The relationship between GM-ITE^®^ scores and resident-level information using multilevel analysis.

| **Factors** | Adjusted estimated coefficient  (95%CI) | p-value |
| --- | --- | --- |
| **Region of Medical School Graduation** |  |  |
| Japan | Reference |  |
| Non-Japan Asia | -1.49 (-4.44 to 1.45) | p = 0.319 |
| Europe and Others | 0.78 (-2.46 to 4.02) | p = 0.635 |
| **Gender** |  |  |
| Women | Reference |  |
| Men | -0.39 (-1.64 to 0.87) | p = 0.546 |
| **Grade** |  |  |
| PGY-1 | Reference |  |
| PGY-2 | 1.73 (0.44 to 3.02) | p = 0.009 |
| **Age** |  |  |
| 24 years old | Reference |  |
| 25 years old | 1.38 (-1.92 to 4.68) | p = 0.411 |
| 26 years old | -0.48 (-3.73 to 2.78) | p = 0.773 |
| 27 years old | -2.69 (-6.10 to 0.71) | p = 0.121 |
| 28 years old | -2.37 (-5.83 to 1.10) | p = 0.180 |
| 29 years old | -1.50 (-5.66 to 2.67) | p = 0.481 |
| Older than 30 years old | -3.64 (-7.09 to -0.20) | p = 0.038 |
| **Night shifts per month** |  |  |
| 0 | Reference |  |
| 1-2 | 1.95 (-0.65 to 4.56) | p = 0.142 |
| 3-5 | 1.39 (-1.08 to 3.87) | p = 0.268 |
| ≥ 6 | 2.54 (-1.27 to 6.35) | p = 0.191 |
| Unknown | -0.95 (-13.37 to 11.47) | p = 0.881 |
| **Average number of assigned inpatients** |  |  |
| 5-9 | Reference |  |
| 0-4 | -1.00 (-2.50 to 0.51) | p = 0.193 |
| 10-14 | -1.37 (-3.10 to 0.36) | p = 0.121 |
| ≥ 15 | -0.59 (-3.44 to 2.27) | p = 0.686 |
| Unknown | -1.29 (-5.58 to 2.99) | p = 0.553 |
| **Self-study time per day (minutes)** |  |  |
| 0 | Reference |  |
| 1-30 | -0.60 (-4.29 to 3.09) | p = 0.749 |
| 31-60 | 1.39 (-2.35 to 5.14) | p = 0.465 |
| 61-90 | 1.91 (-2.16 to 5.99) | p = 0.357 |
| ≥ 91 | 2.22 (-3.27 to 7.72) | p = 0.427 |
| **Duty-hours per week (hours)** |  |  |
| < 45, n (%) | Reference |  |
| ≥45–<50, n (%) | 1.18 (-1.25 to 3.61) | p = 0.339 |
| ≥50–<55, n (%) | 0.58 (-1.93 to 3.09) | p = 0.650 |
| ≥55–<60, n (%) | 1.82 (-0.86 to 4.51) | p = 0.182 |
| ≥60–<65, n (%) | 2.44 (-0.43 to 5.30) | p = 0.095 |
| ≥65–<70, n (%) | 0.77 (-2.34 to 3.89) | p = 0.626 |
| ≥70–<80, n (%) | 1.66 (-1.28 to 4.61) | p = 0.267 |
| ≥80–<90, n (%) | 1.99 (-1.21 to 5.18) | p = 0.222 |
| ≥90–<100, n (%) | 0.08 (-4.32 to 4.49) | p = 0.970 |
| ≥100, n (%) | 2.66 (-2.08 to 7.40) | p = 0.271 |
